# The Pay of the Nurse. VI.—The Lack of Superannuation
*Previous articles appeared March 5, 12, 26, and April 2 and 9.


**Published:** 1921-04-16

**Authors:** 


### THE MATRONS' AND SISTERS' DEPARTMENT.

### THE PAY OF THE NURSE.*

### VI.—The Lack of Superannuation.

No stronger inducement to enter a profession has ever been devised than that afforded by the prospect of security in old age. In whatever direction public authorities stand in need of a large whole-time body of public servants, the promise of a superannuation allowance after a certain number of years' service proves the great attraction. It serves other purposes besides that of attracting the best type of worker. For without superannuation grave distress and difficulties are liable to assail the members of any profession, however well paid. Thrift alone cannot secure the future of any large body of men or women as a whole. Without superannuation, moreover, every service becomes clogged with members who are incompetent, largely by reason of age, and the general standard is lowered in consequence.

The reasons so often urged against the bestowal of superannuation pensions on nurses are familiar to all who have studied this wide question. Nurses receive more than they need for their actual everyday expenses, and ought to lay by for the future. Many do so, and enjoy pensions bought out of the fruit of their labours; it is the extravagant or incompetent who fall into poverty. Nurses are unbusinesslike, and often lose their money by bad speculations, even when they save, etc., etc. The same arguments used to be adduced against inaugurating old-age pensions, and, strangely enough, it was often the retired colonel or Civil Servant in receipt of a pension who was convinced that every labourer who brought up a family on 12s. a week could save for his old age, *if he chose.*

There are definite difficulties in bringing all nurses into a system of superannuation. They do not take kindly to service in one place and under one set of conditions all their lives. Often there is an interlude of marriage and motherhood, after which they resume nursing work. They pass from one branch of nursing to another, from the hospital to the nursing home, from district back again to private work, and from service under public authorities to that of private or philanthropical employers. How can these conditions be reconciled in the matter of superannuation?

To deal with the Nursing Services in all their numerous ramifications in such a way that all nurses could be sure of a pension according to the length of time they devoted to the work it would be necessary to establish a central pension system, financed, let us say, partly by the Health Ministry, under which the Nursing Services generally are grouped, and partly by the private, municipal, philanthropical, and other employers of nurses. In reckoning the value of pensions allowance would be made for the number of duly registered years' service put in by the nurse, beyond a certain minimum, in various branches of her profession.

Private nurses have a very strong claim to participate in any scheme which may be devised for superannuation. There is no denying that it is to the public interest that a far larger number of nurses should be available for this branch of work than can find remunerative employment under normal conditions. The sickness rate fluctuates enormously, and it is not fair that the nurses whose income ceases in times when it is low should bear the whole burden of this unavoidable fluctuation, and be expected to go on saving as usual when out of employment in preparation for the dark days of lessened fees, which begin to loom in sight at forty-five or earlier.

No scheme can be reckoned satisfactory which depends for its success on a voluntary contribution from nurses. If contributory, it must be compulsory. Only so can the danger be avoided of letting just the women most likely to need help in old age slip into a slough of indigence. It should be possible for all nurses to qualify for a small pension by putting in a required number of years' service, after which every year would add to the amount to which she was entitled.

This country will never get the kind of Nursing Service it needs until nursing comes to be regarded as a branch of the national service. The immediate danger is that the women most likely to make good nurses are holding back for lack of adequate inducements to accept this career. The initiation of a pension scheme which does not unduly curtail liberty of action would at once pave the way for a contented and prosperous Nursing Service, and would enormously stimulate thrift among the rank and file, who are only too prone to imagine that saving under present conditions is "no good."

### ROUND THE HOSPITALS.

* Previous articles appeared March 5, 12, 26, and April 2 and 9.

